# Disentangling the effects of self-control and the use of tobacco and cannabis on violence perpetration from childhood to early adulthood

**DOI:** 10.1007/s00787-024-02536-1

**Published:** 2024-07-31

**Authors:** Michelle Loher, Annekatrin Steinhoff, Laura Bechtiger, Denis Ribeaud, Manuel Eisner, Lilly Shanahan, Boris B. Quednow

**Affiliations:** 1 Jacobs Center for Productive Youth Development, University of Zurich, Andreasstrasse 15, P.O. Box 12, Zurich, 8050 Switzerland; 2 University Hospital of Child and Adolescent Psychiatry and Psychotherapy, University of Bern, Bolligenstrasse 111, Bern, 3000, 60 Switzerland; 3 Institute of Criminology, University of Cambridge, Sidgwick Avenue, Cambridge, CB3 9DA UK; 4 Department of Psychology, University of Zurich, Binzmühlestrasse 14, Box 1, Zurich, 8050 Switzerland; 5 Experimental and Clinical Pharmacopsychology, Department of Adult Psychiatry and Psychotherapy, University Hospital of Psychiatry Zurich, University of Zurich, Lenggstrasse 31, PO Box 1931, Zurich, 8032 Switzerland; 6 Neuroscience Center Zurich, University of Zurich and Swiss Federal Institute of Technology, Winterthurerstrasse 190, Y55 J04, Zurich, 8057 Switzerland

**Keywords:** Self-control, Tobacco use, Cannabis use, Violence, Adolescence, Development

## Abstract

Associations among self-control, substance use (e.g., tobacco and cannabis use), and violence perpetration have been documented during the adolescent years, but the direction of these associations is not well understood. Using five assessments (covering 9 years) from a prospective-longitudinal study, we examined self-control as a precursor and subsequent mechanism of associations between adolescent substance use and physical violence perpetration. Data came from a large, ethnically diverse sample (*n* = 1,056). Youth reported their self-control at ages 11, 13, 15, 17, and 20; and their tobacco and cannabis use, and physical violence perpetration at ages 13, 15, 17, and 20. Cross-lagged panel analyses examined associations between these constructs over time. More self-control in late childhood and early adolescence was associated with less future tobacco and cannabis use and physical violence perpetration. Tobacco use was partially associated with more physical violence over time; these associations were not mediated by self-control. Tobacco use in early adolescence was associated with future cannabis use; during late adolescence, tobacco and cannabis use were reciprocally associated over time. Cannabis use was not associated with future physical violence perpetration. Early adolescent self-control plays an important role in later substance use and violence perpetration, and tobacco use has unique links with both later cannabis use and violence perpetration. Supporting the capacities for self-control in late childhood and early adolescence and preventing the initiation and use of entry-level substances could play an important role in preventing both substance use and violence perpetration and their many costs to society.

**Supplementary Information:**

The online version contains supplementary material available at 10.1007/s00787-024-02536-1.

## Introduction

Adolescence is a developmental period characterized by immature self-control and increased sensation-seeking and risk-taking behaviors [1, 2]. For example, substance use, including tobacco and cannabis use, increases during the adolescent years [3, 4]. Adolescence also constitutes the peak lifetime period of violence involvement [5]. Both substance use and violence are very costly to individuals and society [6, 7], and a better understanding of how substance use and violence perpetration precede one another would inform preventions and interventions in important ways. However, although links of alcohol use with subsequent violence perpetration have been established [8], less is known about whether and how tobacco and cannabis use precede violence perpetration specifically during the adolescent years.

Substance use and violence perpetration are both preceded by and associated with lower self-control, which refers to a person’s ability to regulate their behavior (e.g., inhibit impulses), cognition (e.g., defer gratification), and emotions (e.g., exert control over negative emotions) [9, 10]. Self-control emerges in early childhood and, although still diminished during the adolescent years, subsequently becomes more sophisticated [11, 12]. Indeed, variations in levels of self-control predict the onset of both substance use and violence perpetration [13]. Self-control could, however, also be impacted by adolescent substance use.

Specifically, adolescence constitutes a highly neuroplastic period of brain maturation, and tobacco and cannabis could have neurotoxic effects on brain regions and functions associated with self-control [14, 15]. Accordingly, reduced self-control could be a mediator between tobacco or cannabis exposure and subsequent violence perpetration. We review the literature in more detail in the following section.

### Associations of tobacco and cannabis use with physical violence perpetration

Positive associations between tobacco and cannabis use and violence perpetration have been documented [16–20]. However, this work is not always informative about the direction of the effects. Some longitudinal studies have reported that tobacco and cannabis use may precede violent behavior [18, 21, 22]. Other works have suggested the potential reverse direction of effects [23–25], and still other research has reported bidirectional longitudinal associations, especially for cannabis use [26, 27].

These inconsistencies in findings may be due to a number of reasons. First, most of the studies did not feature multiple assessments of tobacco or cannabis use and violence perpetration over the adolescent period. Without repeated assessments during adolescence, the direction of the associations cannot be well understood. Second, previous work typically did not assess physical violence perpetration specifically; instead, it often employed broader measures of aggression and antisocial behavior [23, 24, 28]. Thus, it is unclear whether findings from that work apply specifically to physical violence perpetration. Finally, previous work typically examined males, and, thus, was mostly uninformative about females [22, 28]. Taken together, the direction of the association of tobacco/cannabis use with violence perpetration needs to be examined with longer-term longitudinal studies of males and females and with specific assessments of physical violence perpetration.

### Does self-control predict substance use and physical violence perpetration and mediate their subsequent associations?

The mechanisms underlying the associations between tobacco and cannabis use and violence perpetration are not fully understood; self-control is a likely candidate. First, low self-control may precede adolescent substance use and violent behaviors. Indeed, several studies have shown that low self-control in childhood predicts later aggression, antisocial behavior, substance use, and addictive behavior in adolescence [29, 30]. Second, when substance use and violent behavior have been initiated, diminished self-control could mediate their subsequent association. Particularly, tobacco and cannabis could exert neurotoxic effects on the developing adolescent brain, including in regions and functions governing self-control [14, 15]. In other words, there may be associations between adolescent tobacco and cannabis consumption and certain brain changes that are related to self-control, which, in turn, could be linked to an increased likelihood of physical violence perpetration. Taken together, low self-control could initially be a precursor of substance use and physical violence perpetration, and, subsequently, a mediator of associations between the two.

**Fig. 1:**
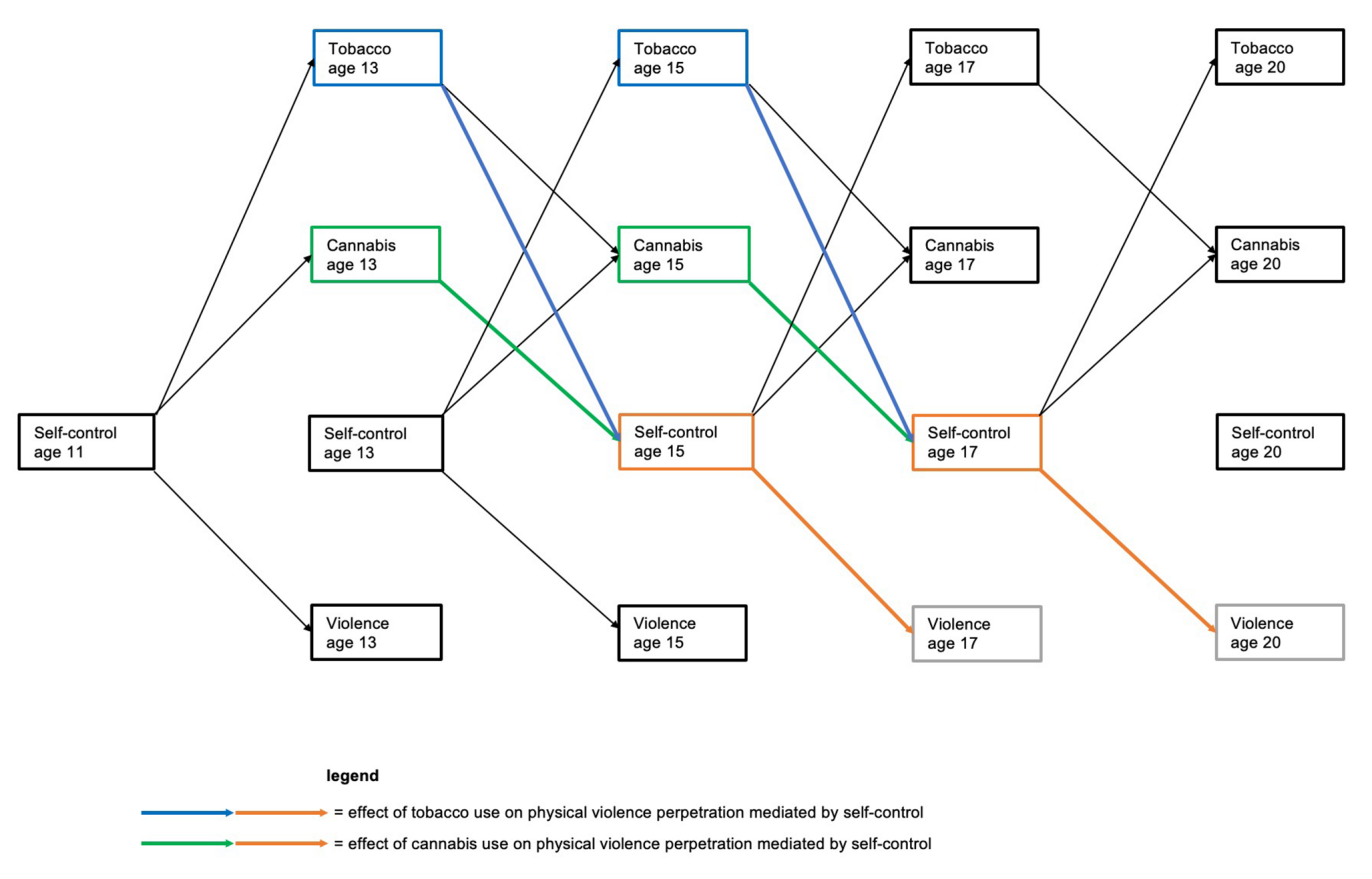
Hypothesized associations among self-control, tobacco and cannabis use, and physical violence perpetration. For the sake of simplicity, the direct paths between tobacco use and physical violence perpetration, those between cannabis use and physical violence perpetration, and the stabilities of constructs over time are not shown in the figure. Instead, we show the potential indirect effects via self-control and the direct paths between tobacco and cannabis use, and the direct paths of self-control with tobacco and cannabis use, and physical violence perpetration

Here, we illuminate the complex longitudinal interplay among self-control, tobacco and cannabis use, and physical violence perpetration from late childhood to early adulthood in a large population-based sample. Specifically, we aim to test self-control as a precursor of and a mechanism through which tobacco and cannabis use are linked with physical violence perpetration over time (see Fig. 1). We expected that high self-control in late childhood and early-to-late adolescence would be associated with less frequent tobacco and cannabis use and less physical violence perpetration in early-to-late adolescence and young adulthood, and that subsequent pathways from substance use to physical violence perpetration would be explained in part by lower self-control (i.e., indirect effects of tobacco and cannabis use at age 13 and 15 on physical violence perpetration at age 17 and 20 respectively through self-control at age 15 and 17). In addition, consistent with the Gateway Hypothesis—that is, certain substances provide gateways to the use of other substances—we expected that earlier tobacco use would be associated with future cannabis use [31]. Tobacco use is often initiated during adolescence, and typically precedes the use of other (illegal) substances. Indeed, in 2011 (i.e., when our participants were 13 years old), tobacco was an entry-level substance in Switzerland [32].

## Methods

### Participants and procedure

Data were drawn from the Zurich Project on the Social Development from Childhood to Adulthood (z-proso), a prospective longitudinal study [33]. The target population consisted of all first graders in Zurich’s public primary schools in 2004. Children (*N* = 1,675) from 56 schools were randomly selected using a stratified sampling method (slight oversampling from disadvantaged school districts). Further details on the sample, assessment procedures, and attrition can be found in previous work [34]. At the time of writing, the eight main z-proso survey waves at ages 7, 8, 9, 11, 13, 15, 17, and 20 years were available for analysis. Data used in the present study were collected at ages 11, 13, 15, 17, and 20 years. Consistent with Zurich’s diverse population, participants’ parents were born in > 80 different countries (see [35]), but most of the youth participants were born in Switzerland.

The age 11 to age 17 assessments were conducted in public school classrooms with paper-and-pencil questionnaires (requiring approx. 90 min to complete). At age 20, participants completed computer-aided questionnaires in a computer laboratory setting. Participants received financial compensation for their time (∼ 30 USD at age 13 to ∼ 75 USD at age 20).

*N* = 1,583 (94.5%) of the 1,675 originally targeted for participation contributed data at one or more study assessments via at least one informant (child, parent, or teacher.) At the timepoints relevant for our analyses, the participation rate was as follows: *n* = 1,143 (68.2% of target sample; or 72,2% of those who participated on the study at least once via one informant) at age 11, *n* = 1,365 (81.5% of target sample) at age 13, 1,446 (86.3% of target sample) at age 15, *n* = 1,306 (78.0% of target sample) at age 17, and, *n* = 1,180 (70.4% of target sample) at age 20. At age 15, z-proso had its highest participation rate. Previous work documents detailed attrition levels in this sample [36, 37]. Participants with at least one Swiss-born parent were more likely to participate in the age 20 assessment compared to those whose parents were both born abroad (83.9% vs. 77.9%, *p* = .004) [37]. In addition, respondents at the age 20 assessment had a higher family socioeconomic status (SES) during adolescence than those who had dropped out (ISEI score: *M* = 47.1 [*SD* = 19.7] vs. *M* = 40.4 [*SD* = 16.6], *p* < .001) [37].

### Measures

*Self-control* was self-reported from ages 11 to 20 on 10 items adapted from the self-control scale [38, 39]. The scale assesses risk-seeking, impulsivity, self-centeredness, preference for physical activities, and short temperedness/low frustration tolerance. The response options ranged from 1 = *false* to 4 *= true*. For the present study, items were reverse-coded, with higher scores indicating higher self-control. The reliability and validity of the measure has been supported by previous research in the current sample [40]. In our study, Cronbach’s α estimated with the analytical sample ranged from 0.72 to 0.77 from ages 11 to 20. For additional information, see online Supplementary Appendix A.

*Tobacco use* and *cannabis use* frequency over the past 12 months were each self-reported on a six-point Likert scale with one item from ages 13 to 20. The response options were 1 (“never”), 2 (“once”), 3 (“2 to 5 times”), 4 (“6 to 12 times [monthly]”), 5 (“13 to 52 times [weekly]”), and 6 (“53 to 365 times [daily]”). *Alcohol use* frequency over the past 12 months was also self-reported from ages 13 to 20 and included in a sensitivity analysis (for details, see online Supplementary Appendix A).

*Physical violence perpetration* in the past 12 months was operationalized using three self-reported items. Two items came from the Social Behaviour Questionnaire (SBQ) [41]: violent attack and engagement in physical fights or brawls were each rated from 1 = *never* to 5 = *very often*. An additional physical assault item came from a peer aggression questionnaire [42] and was rated from 1 = *never* to 6 = *(almost) every day*. Z-scores were computed for the items from the two scales separately and then averaged for each assessment. Cronbach’s α estimated with the analytical sample ranged from 0.68 to 0.81 from ages 13 to 20.

*Control variables* included *sex assigned at birth* (dummy coded: 1 = male, 0 = female), *parental migration background* (dummy coded: 1 = both parents born abroad, 0 = at least one parent born in Switzerland), and *family socioeconomic status* (SES). The latter was assessed according to the International Socio-Economic Index of Occupational Status (ISEI) [43], an index based on occupation-specific income and required educational level [min. score of 16 (e.g., unskilled worker) and max. score of 90 (e.g., judge)]. The highest ISEI score of the two caregivers between the participants’ ages of 11 and 15 was used to maximize the *n* on this variable.

### Statistical analyses

First, we assessed bivariate correlations among the study variables. Second, we conducted cross-lagged panel analyses within a structural equation modeling framework to examine the prospective associations among self-control, tobacco use, cannabis use, and physical violence perpetration. Cross-lagged panel models (CLPM) allow examination and description of the reciprocal associations of different variables over time [44]. The models used here do not account for trait-like between-person differences in the observed variables, as the complexity of our models (e.g., four main variables assessed at multiple timepoints and inclusion of numerous control variables) led to convergence issues [45].

We estimated autoregressive effects, cross-lagged effects, and (residual) variances. Autoregressive and cross-lagged effects were specified for all constructs and between all construct at adjacent time points. In addition, direct associations across larger lags were included from tobacco and cannabis use at age 13 to physical violence perpetration at ages 17 and 20 respectively, as well as from tobacco and cannabis use at age 15 to physical violence perpetration at age 20 to adequately estimate the indirect effects. Residuals of constructs assessed at the same survey wave were allowed to covary. Given that adolescence is characterized by many changes [4], we allowed the cross-lagged and autoregressive paths to vary freely over time.

To test the indirect effects of the examined substances (i.e., tobacco, cannabis) on physical violence perpetration through self-control, a bias-corrected bootstrapping procedure (10,000 draws) was used. This procedure estimates accurate confidence intervals for indirect effects [46]. Maximum likelihood estimation was used for the overall model analysis.

Alcohol is another commonly used substance during adolescence and the co-use with cannabis is prevalent [47]. Accordingly, we conducted a sensitivity analysis to investigate the potential confounding effect of alcohol use in an additional CLPM that replaced tobacco use with alcohol use.

The models were estimated in R 4.2.2 [48] using the “lavaan” package for structural equation modeling [49]. Full information maximum likelihood estimation (FIML) was used to reduce possible bias due to attrition mechanisms [50], and all coefficients were standardized [51]. Since the $\:{\upchi\:}$^2^ fit statistic is very sensitive with large sample sizes [52], the following criteria were used to evaluate the model fit: the Comparative Fit Index (CFI) and the Tucker Lewis Index (TLI), both indicating adequate model fit if > 0.90, as well as Root Mean Squared Error of Approximation (RMSEA) and Standardized Root Mean Square Residuals (SMSR), both indicating adequate model fit if < 0.05 [53]. Control variables (sex, parental migration background, family SES) were included as predictors of all endogenous variables in a first step and were then excluded if non-significant (*p* > .10) for model parsimony. The specification of covariates as exogenous variables led to the exclusion of cases with missing values for these controls. Self-control assessed at age 11 was also specified as an exogenous variable. In total, 1,056 cases were used for all the analyses reported in this paper.

## Results

### Descriptive statistics

Among the 1,056 participants, 51.14% were male. A slight majority reported having at least one parent born in Switzerland (55.49%) and the mean family SES was 47.81 (SD = 19.7). At age 13, approximately one in four had used tobacco in the past year and almost 10% had used cannabis in the previous year. At age 15, 60% of the participants (*n* = 617) had reported tobacco use during the past year, 35% reported having used cannabis in the past year. Frequent cannabis use (i.e., weekly or daily use) was near 10% at age 15. For a comprehensive overview of the prevalence rates of substance use (covering past-year prevalence and frequent use) see Online Supplement (Figure S1a-b).

Correlations and summary statistics of all study variables are shown in Table 1. Self-control, tobacco use, cannabis use, and physical violence perpetration were significantly correlated from late childhood to early adulthood. Self-control was negatively associated with substance use and physical violence perpetration. Tobacco use was positively associated with cannabis use; and both substances were also positively associated with physical violence perpetration.

**Table 1:** Correlations, means, and standard deviations of all study variables

Variables	1	2	3	4	5	6	7	8	9	10	11	12	13	14	15	16	17	*n*	M (SD)
1. Self-control _age11_	1																	1056	3.05(0.47)
2. Self-control _age13_	0.44^***^	1																979	2.80(0.47)
3. Self-control _age15_	0.33^***^	0.52^***^	1															1035	2.73(0.44)
4. Self-control _age17_	0.32^***^	0.43^***^	0.62^***^	1														947	2.79(0.42)
5. Self-control _age20_	0.23^***^	0.33^***^	0.46^***^	0.60^***^	1													879	2.94(0.42)
6. Tobacco use _age 13_	−0.22^***^	−0.36^***^	−0.23^***^	−0.20^***^	−0.15^***^	1												968	1.61(1.19)
7. Tobacco use _age 15_	−0.22^***^	−0.36^***^	−0.35^***^	−0.27^***^	−0.19^***^	0.52^***^	1											1029	2.73(1.75)
8. Tobacco use _age 17_	−0.20^***^	−0.30^***^	−0.35^***^	−0.35^***^	−0.24^***^	0.38^***^	0.64^***^	1										946	3.48(1.88)
9. Tobacco use _age 20_	−0.12^***^	−0.22^***^	−0.26^***^	−0.29^***^	−0.25^***^	0.30^***^	0.49^***^	0.67^***^	1									878	3.63(1.9)
10. Cannabis use _age13_	−0.27^***^	−0.25^***^	−0.17^***^	−0.14^***^	−0.15^***^	0.54^***^	0.30^***^	0.22^***^	0.14^***^	1								968	1.18(0.67)
11. Cannabis use _age15_	−0.22^***^	−0.32^***^	−0.28^***^	−0.23^***^	−0.17^***^	0.41^***^	0.60^***^	0.40^***^	0.28^***^	0.38^***^	1							1027	1.93(1.46)
12. Cannabis use _age17_	−0.26^***^	−0.29^***^	−0.24^***^	−0.29^***^	−0.18^***^	0.30^***^	0.43^***^	0.49^***^	0.38^***^	0.29^***^	0.57^***^	1						940	2.54(1.67)
13. Cannabis use _age20_	−0.20^***^	−0.25^***^	−0.20^***^	−0.24^***^	−0.28^***^	0.24^***^	0.28^***^	0.35^***^	0.43^***^	0.21^***^	0.40^***^	0.61^***^	1					878	2.56(1.68)
14. Violence _age13_	−0.26^***^	−0.44^***^	−0.30^***^	−0.26^***^	−0.24^***^	0.37^***^	0.31^***^	0.24^***^	0.20^***^	0.33^***^	0.26^***^	0.23^***^	0.22^***^	1				982	−0.01(0.81)
15. Violence _age15_	−0.19^***^	−0.34^***^	−0.38^***^	−0.29^***^	−0.25^***^	0.30^***^	0.35^***^	0.23^***^	0.15^***^	0.24^***^	0.26^***^	0.20^***^	0.17^***^	0.50^***^	1			1037	−0.03(0.79)
16. Violence _age17_	−0.19^***^	−0.21^***^	−0.23^***^	−0.33^***^	−0.23^***^	0.20^***^	0.24^***^	0.25^***^	0.18^***^	0.21^***^	0.20^***^	0.19^***^	0.14^***^	0.34^***^	0.51^***^	1		954	−0.04(0.81)
17. Violence _age20_	−0.11^**^	−0.09^**^	−0.17^***^	−0.21^***^	−0.31^***^	0.15^***^	0.19^***^	0.20^***^	0.20^***^	0.18^***^	0.14^***^	0.08^*^	0.13^***^	0.21^***^	0.36^***^	0.41^***^	1	880	−0.02(0.74)
*Controls*																			
Sex (1)	−0.17^***^	−0.14^***^	−0.08^**^	−0.11^***^	−0.14^***^	0.12^***^	0.05	−0.01	0.00	0.21^***^	0.18^***^	0.17^***^	0.19^***^	0.36^***^	0.32^***^	0.35^***^	0.23^***^	1056	51%
Migration background (1)	0.11^***^	0.05	−0.02	−0.03	−0.05	−0.02	0.00	0.01	0.04	−0.03	−0.15^***^	−0.19^***^	−0.17^***^	0.08^*^	0.07^*^	0.10^**^	0.11^**^	1056	45%
Family SES	−0.08^**^	−0.02	0.06	0.10^**^	0.11^**^	−0.03	−0.03	−0.05	−0.09^**^	−0.01	0.15^***^	0.19^***^	0.15^***^	−0.13^***^	−0.12^***^	−0.14^***^	−0.14^***^	1056	47.81(19.7)

*Note. N* = 1,056, *M* = mean, *SD* = standard deviation; *p < .05, **p < .01, ***p < .001. Missing = pairwise. Dummy coding: Sex assigned at birth (1) = male; parental migration background (1) = both parents born abroad

### Cross-lagged panel analysis

**Fig. 2:**
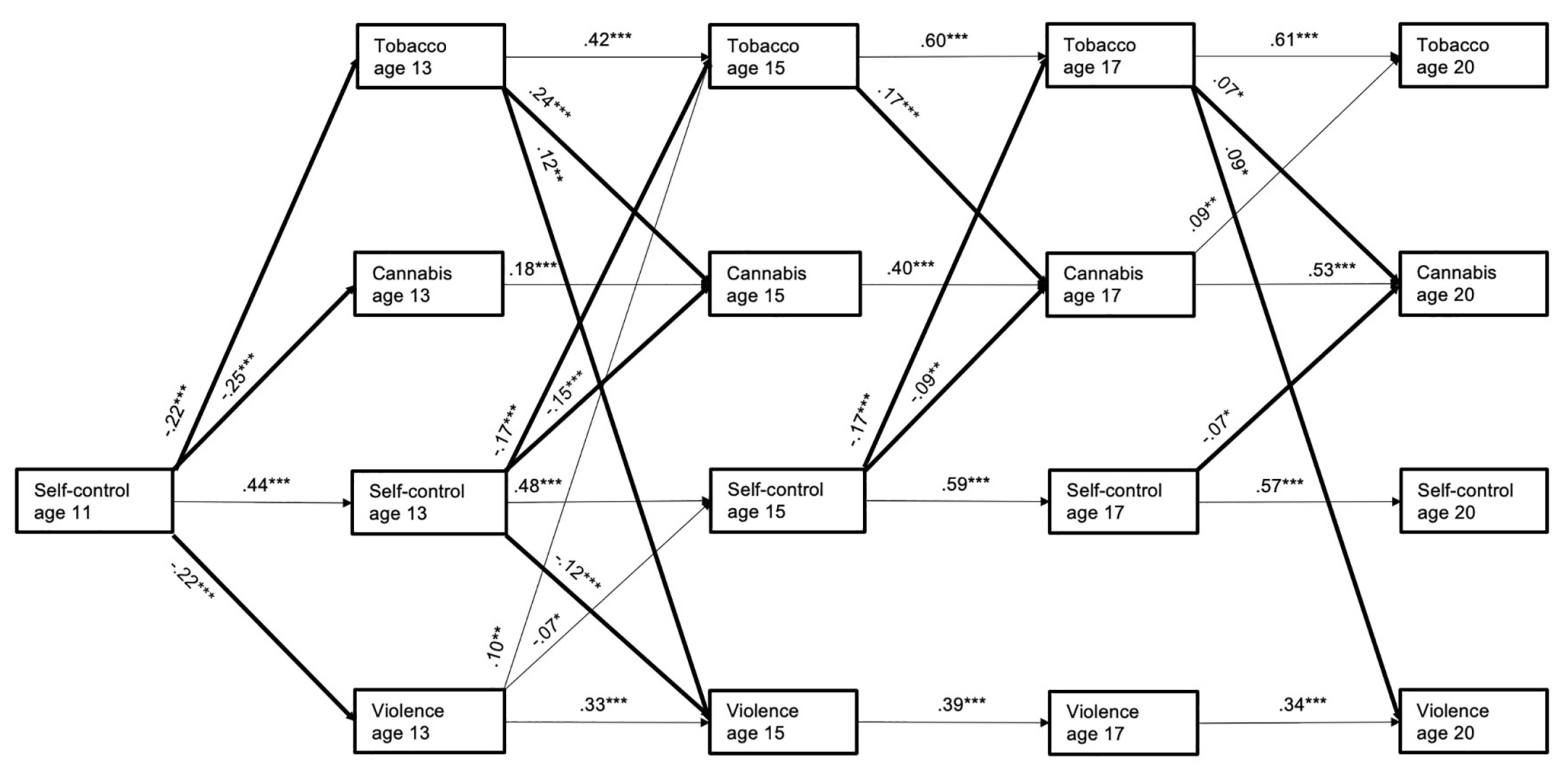
Cross-lagged panel model connecting self-control, tobacco use, cannabis use, and physical violence perpetration. *N* = 1,056 *p < .05, **p < .01, ***p < .001. Significant standardized path coefficients. Bold arrows support the hypotheses. For the sake of simplicity, cross-sectional covariances of residuals are omitted here, but they are shown in the Online Supplement, Table S2. The model is adjusted for sex assigned at birth, parental migration background, and family SES

The model yielded a good fit: $\:{\upchi\:}$^2^ (75) = 168.766, *p* < .001; CFI = 0.986; TLI = 0.966; RMSEA = 0.034; SRMR = 0.028. The significant standardized coefficients for autoregressive and cross-lagged paths are presented in Fig. 2, and unstandardized coefficients are shown in the Online Supplement (Table S1). The autoregressive paths (i.e., stability coefficients) of the main study variables were significant across all time points.

In terms of cross-lagged paths, self-control in late childhood and early adolescence (ages 11 and 13) was significantly negatively associated with substance use and physical violence perpetration at ages 13 and 15 respectively; this effect extended to age 20 for cannabis use. Tobacco use at ages 13 and 17 was significantly positively associated with physical violence perpetration at the next assessment. In turn, age 13 physical violence perpetration was also weakly linked to more tobacco use at age 15. Consistent with the Gateway Hypothesis [31], tobacco use was also positively associated with future cannabis use across all time points. A posthoc wald test showed this association was strongest in early adolescence and weakened with increasing age. During late adolescence (age 17), cannabis use, in turn, was also positively associated with tobacco use at age 20. Physical violence perpetration at age 13 was weakly associated with lower self-control at age 15. Finally, cannabis use and physical violence perpetration were not significantly associated across time.

Covariances of residuals were significant, except for cross-sectional covariances of residuals of cannabis use and physical violence perpetration at ages 17 and 20 (see online Supplementary Table S2 for coefficients).

In terms of the control variables, being male was positively associated with physical violence perpetration and cannabis use across all timepoints and with tobacco use at age 13 (see online Supplementary, Table S3 for coefficients). In addition, being male was negatively associated with self-control at ages 13, 17, and 20. Parental migration background showed a weak positive association with physical violence perpetration at age 13. Furthermore, youth with a parental migration background were less likely to use cannabis in mid and late adolescence and early adulthood, whereas those with higher SES were more likely to consume cannabis in mid and late adolescence. A higher SES was negatively associated with tobacco use in young adulthood. In addition, youth with a higher SES were less likely to engage in physical violence perpetration at all time points.

#### Mediation analysis

The mediation analysis estimated the indirect pathways depicted in Table 2 between both substances (i.e., tobacco and cannabis) and physical violence perpetration through self-control. Each confidence interval includes 0, meaning that none of the hypothesized indirect effects were significant. In other words, self-control had direct effects on all other outcomes, but it was not a mediating factor in the associations of substance use with physical violence perpetration, as it was not associated with prior substance use.

**Table 2:** Unstandardized estimates of Indirect effects, Standard Errors, and 95% Bias-corrected bootstrap confidence intervals

				Confidence intervals	
	Estimate	SE		Lower	Upper
T_age 13_→ SC_age 15_ → V_age 17_ → V_age 20_	0.000	0.001		0.000	0.002
T_age 13_→ SC_age 15_ → V_age 17_	0.001	0.002		−0.001	0.006
T_age 13_→ T_age 15_ → SC_age 17_ → V_age 20_	0.000	0.001		0.000	0.002
T_age 15_ → SC_age 17_ → V_age 20_	0.001	0.001		−0.001	0.004
T_age 13_→ SC_age 15_ → SC_age 17_ → V_age 20_	0.000	0.001		−0.001	0.003
C_age 13_→ SC_age 15_ → V_age 17_ → V_age 20_	0.000	0.001		−0.001	0.003
C_age 13_→ SC_age 15_ → V_age 17_	0.001	0.003		−0.003	0.009
C_age 13_→ C_age 15_ → SC_age 17_ → V_age 20_	0.001	0.001		0.000	0.003
C_age 15_ → SC_age 17_ → V_age 20_	0.001	0.001		0.000	0.006
C_age 13_→ SC_age 15_ → SC_age 17_ → V_age 20_	0.000	0.002		−0.002	0.005

Note. T = tobacco use, C = cannabis use, SC = self-control, V = physical violence perpetration; *n* = 1,056. Direct associations included cross-lagged paths from tobacco and cannabis use at age 13 to physical violence perpetration at age 17 and age 20 as well as from tobacco and cannabis use at age 15 to physical violence perpetration at age 20

#### Sensitivity analysis

The potential confounding effect of alcohol use was examined in an additional CLPM where alcohol use was included instead of tobacco use. Model estimates can be found in the Online Supplement (Figure S2, Table S5). Cross-lagged paths indicated that for cannabis, the effects remained very similar to those in the main model. Alcohol use at age 15, however, was indirectly associated with more physical violence perpetration at age 20 via self-control at age 17 (see online Supplementary Tables S4–S7).

## Discussion

Drawing on five waves of data from a prospective longitudinal cohort study in urban Switzerland (z-proso) with relatively high rates of early cannabis consumption (e.g., one in three had used cannabis in the previous year at age 15) [54], our study provides new insights into the longitudinal associations of self-control, tobacco and cannabis use, and physical violence perpetration from late childhood to early adulthood.

### Self-control as a predictor of substance use and physical violence perpetration

Our results showed that self-control is a precursor of adolescent substance use and physical violence perpetration. More self-control in late childhood and early adolescence was significantly associated with less future tobacco and cannabis use and less physical violence perpetration to age 15. Contrary to our hypothesis, however, these associations continued solely for cannabis use to age 20.

From a prevention perspective, the findings suggest that supporting adolescents’ capacities for self-control could play a central role in preventing both adolescent substance use and physical violence perpetration during the period when these behaviors are often first initiated. Indeed, previous research has shown that self-control interventions in young people are effective in reducing aggressive and violent behavior [55, 56]. They should ideally be used in the first decade of life [57], as patterns of behaviors become more stable and entrenched thereafter [58]. In our study, self-control was indeed moderately stable from ages 11 to 20. Specific strategies for teaching self-control, including making it part of the regular school curriculum, have recently been recommended [59].

### Substance use and physical violence perpetration, and the role of self-control as a mediator

Consumption of tobacco products in early adolescence may result in neurotoxicity for the developing adolescent brain and lead to sustained neuronal and behavioral alterations [14, 60]. Consistent with this idea, adolescent tobacco use in our sample was associated with physical violence perpetration in early and late adolescence. However, despite moderate to high cross-sectional bivariate correlations between tobacco use and self-control (e.g., *r*_self−control−tobaccoage13_ = − 0.36, *p* < .001), the associations between tobacco use and physical violence perpetration were not mediated by self-control in our model. This might indicate an independent tobacco-driven effect on violence perpetration, whose occurrence across different assessments suggests robustness.

One possible explanation could be peer influence. Indeed, peers play an important role for adolescent development [61], and peer delinquent behavior, including the use of entry-level substances, is considered a general risk factor for delinquency and violent crime [62]. In other words, adolescent tobacco use could occur in the presence of peers with a greater inclination toward violence, which may in turn increase the risk of violence perpetration. This should be examined in future research.

Alternatively, the lack of indirect associations found could be explained by the presence of another mechanism of action, such as a potential shared genetic etiology of substance use and violence perpetration. Genetic predisposition for aggressive and violent behavior might not only be associated with violence itself, but also with substance use [63]. Indeed, previous research has found genetic correlations between predisposition for aggression and smoking phenotypes such as earlier smoking initiation [64].

Also, our self-control measure, which was originally developed for the purpose of criminology research [38], may not have been as sensitive to neural substance-induced changes as other laboratory-based paradigms (e.g., stop-signal tasks) [65, 66]. In fact, findings from our sensitivity analysis with alcohol suggest that self-control mediates some associations between earlier alcohol use and later physical violence perpetration. This is particularly interesting since the impact of adolescent alcohol use on normative brain development remains mostly unknown [67].

Contrary to previous studies, our results suggest that cannabis use is not uniquely associated with future physical violence perpetration when the effects of self-control and tobacco use are taken into account. Also, findings from our sensitivity analysis suggest that, when controlling for the most-used substance, alcohol, cannabis use does not have a unique effect on physical violence perpetration over time. To the best of our knowledge, however, no prior study has examined self-control, tobacco/alcohol use, cannabis use, and physical violence perpetration in one model across the adolescence period. One possible explanation for the nonsignificant paths is the time lag of two to three years between assessments. During highly dynamic periods of development, such as adolescence, large time lags may lead to an underestimation of lagged effects [68]. In addition, overall, the magnitude of the cross-lagged associations in our model was small to moderate in size. This is not surprising, considering that the cross-lagged effects are controlled for prior autoregressive effects (i.e., stability coefficients) of the outcome variable [69].

It is possible that cannabis has acute rather than chronic effects on impulsive and violent behavior [70], which our longer-term longitudinal design would not have been able to capture.

### Developmental associations between tobacco and cannabis use

Our longitudinal data, collected between 2011 and 2018, provide some empirical support for the Gateway Hypothesis [31]; that is, consumption of tobacco products precedes cannabis use. Besides the assumption that nicotine might prime the brain for use of other substances [71], gateway effects could also reflect common liability effects (e.g., individual vulnerability) [72], or the transfer of learning (i.e., learning the rewarding effects of one substance increases the likelihood of instrumentalizing the rewarding effects of a new substance) [73, 74] In addition, drug availability, especially in early adolescence (e.g., through peer affiliations) might also play an important role in the transition from tobacco use to cannabis use. Interestingly, in later adolescence, a reciprocal association was found, suggesting a possible reverse gateway effect [75] from late adolescence to early adulthood. These reciprocal associations are consistent with prior findings [76] and suggest that in a context in which cannabis use is common, like among 17-year-olds in Zurich [54], cannabis use may also precede tobacco use, and even be a contributing factor to its onset. This may partially be explained by the common route of administration, i.e., if smoked with tobacco [77].

Nevertheless, our results add to the understanding of the longitudinal relationship between tobacco and cannabis use and contribute new information to ongoing debates about legalizing cannabis. Switzerland already records high rates of substance use among young people [32] and a legalized cannabis market will likely be implemented within the next years.

### Limitations

First, substance use was assessed via self-reporting, meaning that under- (or over)reporting is a possibility [78]. Second, the questionnaire did not capture newer nicotine-containing products, such as e-cigarettes. However, these were uncommon in Switzerland before 2018 (i.e., when the age 20 assessment took place). Third, cannabis potency was not assessed, although it has changed across the observation period [79]. More potent cannabis products are more likely to increase the risk of psychosis [80], which, in turn, could lead to violence perpetration [81]. Fourth, the co-use of tobacco and cannabis (i.e., smoked together in “joints”) was not explicitly assessed, but it is possible that the neurotoxic effects of the two substances used jointly could reinforce one another. Finally, cross-lagged panel models are powerful tools for establishing pathways of related constructs across adolescence. However, trait-like interindividual differences are encompassed in the autoregressive and cross-lagged paths, which may bias the findings [82].

### Conclusion

Our findings highlight the importance of self-control in late childhood and early adolescence as a predictor of subsequent tobacco and cannabis use and physical violence perpetration. They also show unique associations of adolescent tobacco use with both later cannabis use and physical violence perpetration. Supporting the capacities for self-control in late childhood and early adolescence and preventing adolescent tobacco use could help prevent future physical violence perpetration and future cannabis use and their many costs to individuals and society.

## Electronic supplementary material

Below is the link to the electronic supplementary material.

Supplementary Material 1

## Data Availability

Requests for code can be made through the first author of this publication.
