# Supplementary material for: Disentangling the effects of self-control and the use of tobacco and cannabis on violence perpetration from childhood to early adulthood
Source: Eur Child Adolesc Psychiatry. 2024 Jul 31;34(3):1063–74. doi: 10.1007/s00787-024-02536-1 (PMC11909040; doi:10.1007/s00787-024-02536-1)
Supplement: Supplementary file 1 — Supplementary Material 1 [file 787_2024_2536_MOESM1_ESM.docx]

# Disentangling the effects of self-control and the use of tobacco and cannabis on violence perpetration from childhood to early adulthood

**Supplement**

Michelle Loher, Annekatrin Steinhoff, Laura Bechtiger, Denis Ribeaud, Manuel Eisner, Lilly Shanahan^♣^ & Boris B. Quednow^♣^

^♣^These authors contributed equally.

**Journal name:** European Child & Adolescent Psychiatry

**Corresponding author:**

Michelle Loher, MSc

Jacobs Center for Productive Youth Development

University of Zurich

Andreasstrasse 15, P.O. Box 12

8050 Zurich, Switzerland

E-Mail: [michelle.loher@jacobscenter.uzh.ch](mailto:michelle.loher@jacobscenter.uzh.ch)

# Appendix A. Specification of Variables (Main Model and Sensitivity Analysis)

## Self-Control (adapted from Grasmick et al [1]; later modified by Longshore et al [2])

Below are various statements. Please mark with a cross, how true these statements are *for you.*

- I often act on the spur of the moment without stopping to think.
- I try to get what I want even if it causes problems for others.
- I like to do dangerous things just for the fun of it.
- If I don’t get something I want immediately, I get angry pretty quickly.
- I prefer to go out and do something instead of reading or thinking.
- If others get angry about something I've done, I don't really care.
- When I can, I prefer to do something with my hands rather than my head.
- I always do what I feel like doing at the moment, without thinking about what the consequences might be.
- I lose my temper pretty easily.
- Excitement and adventure are more important to me than safety.

## Substance Use (created by the study team)

How many times in the last 12 months (i.e., since April 2014) have you taken …

- Cigarettes, tobacco, or shisha?
- Hash, “pot,” cannabis, or marijuana?
- Beer, wine, or “mixed drinks”?
- Vodka, whiskey, or schnapps?

*Alcohol.* Alcohol use was measured using two items from a larger set of items assessing different types of self-reported substances. One item assessed the use of *beer/wine/“mixed drinks,”* whereas the other assessed the use of *liquor* (e.g., vodka, whiskey, schnapps). Answer categories concerning the frequency of consumption (in the last 12 months) were provided on a 6-point Likert scale (*never*–*daily*). For each participant, the maximum value from both items was computed (from the age 13, 15, 17, and 20 assessments) and used for analysis.

## Physical Violence Perpetration

### Social Behaviour Questionnaire [3]

Please indicate which of these things you have done *in the last 12 months* (i.e., since April 2014).

- You physically attacked other people.
- You got into fights.

### Peer aggression questionnaire [4]

This part is about bullying. Adolescents can be very mean to each other sometimes. And you? In the last year, i.e., since April 2014, have you bullied other adolescents? (This could have happened, for example, on the way to school, when being out, at home, or on the internet.) How many times since April 2014 have you...

- Hit, bitten, kicked or pulled their hair?


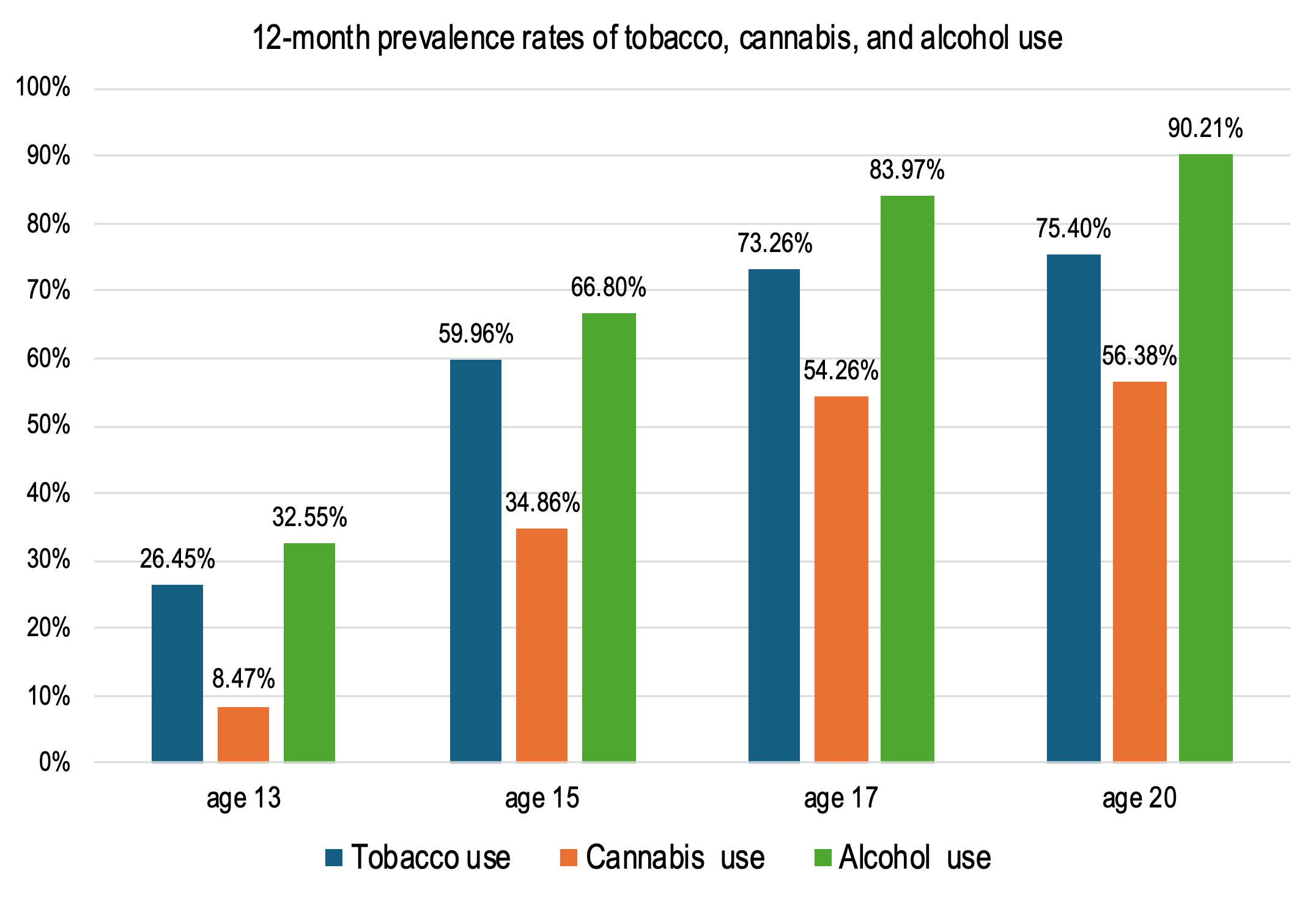


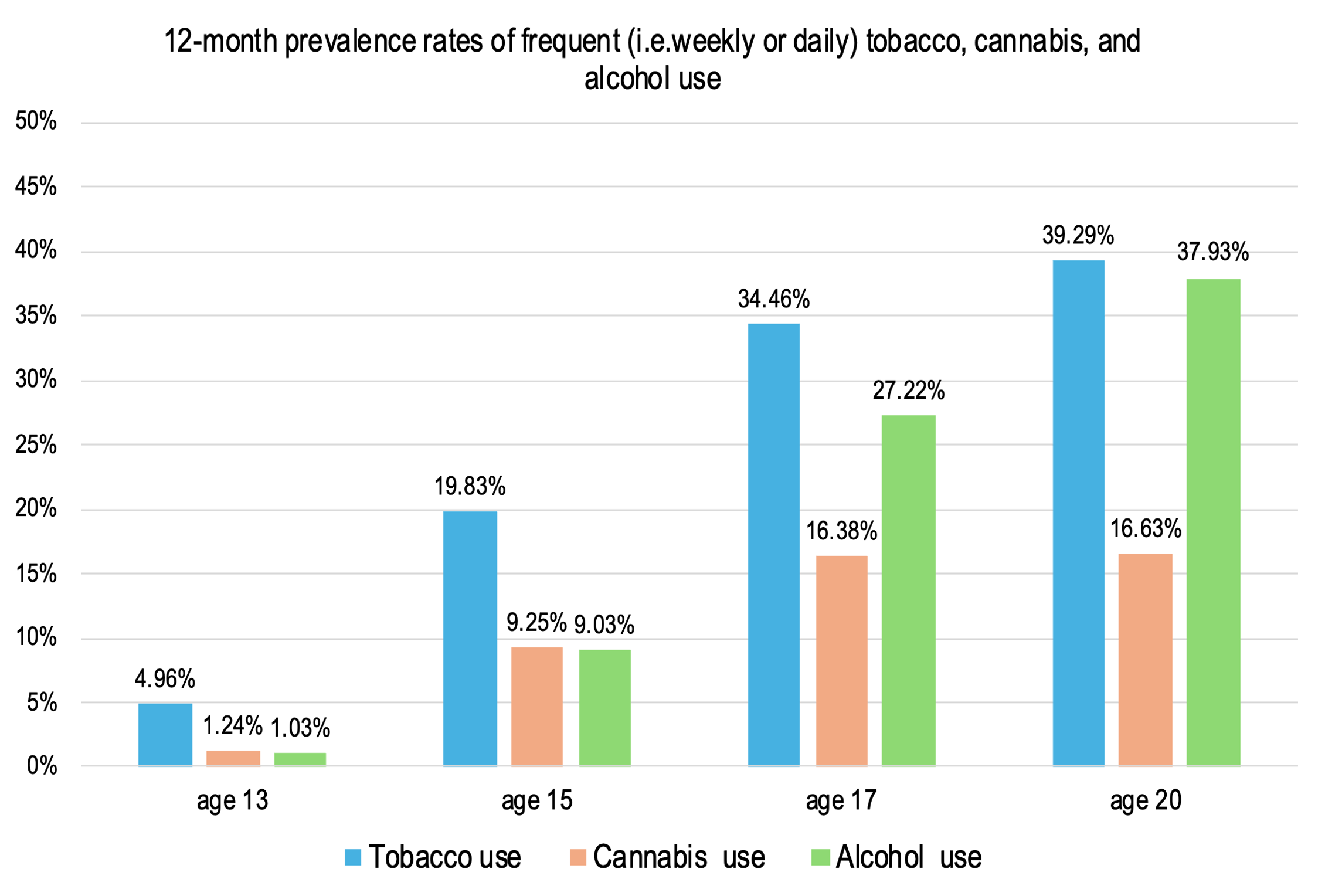


**Fig. S1a-b** 12-month prevalence rates of tobacco, cannabis, and alcohol use. Frequent use refers to weekly or daily use, *n* = 1,056.

# Appendix B. Coefficients Main Model and Sensitivity Analysis

## Main Model

Table S1

Standardized and Unstandardized Coefficients for Autoregressive and Cross-Lagged Paths (Main Model)

| CLPM |  | Unstandardized beta |  | SE |  | *p*-value |  | Standardized beta (β) |
| --- | --- | --- | --- | --- | --- | --- | --- | --- |
| *Autoregressive paths* |  |  |  |  |  |  |  |  |
| Self-control _age 11_ 🡪 Self-control _age 13_ |  | 0.43 |  | 0.03 |  | < .001 |  | 0.44 |
| Self-control _age 13_ 🡪 Self-control _age 15_ |  | 0.45 |  | 0.03 |  | < .001 |  | 0.48 |
| Self-control _age 15_ 🡪 Self-control _age 17_ |  | 0.56 |  | 0.03 |  | < .001 |  | 0.59 |
| Self-control _age 17_ 🡪 Self-control _age 20_ |  | 0.57 |  | 0.03 |  | < .001 |  | 0.57 |
| Tobacco use _age 13_ 🡪 Tobacco use _age 15_ |  | 0.61 |  | 0.05 |  | < .001 |  | 0.42 |
| Tobacco use _age 15_ 🡪 Tobacco use _age 17_ |  | 0.65 |  | 0.03 |  | < .001 |  | 0.60 |
| Tobacco use _age 17_ 🡪 Tobacco use _age 20_ |  | 0.62 |  | 0.03 |  | < .001 |  | 0.61 |
| Cannabis use _age 13_ 🡪 Cannabis use _age 15_ |  | 0.40 |  | 0.10 |  | < .001 |  | 0.18 |
| Cannabis use _age 15_ 🡪 Cannabis use _age 17_ |  | 0.46 |  | 0.05 |  | < .001 |  | 0.40 |
| Cannabis use _age 17_ 🡪 Cannabis use _age 20_ |  | 0.54 |  | 0.04 |  | < .001 |  | 0.53 |
| Violence _age 13_ 🡪 Violence _age 15_ |  | 0.33 |  | 0.05 |  | < .001 |  | 0.33 |
| Violence _age 15_ 🡪 Violence _age 17_ |  | 0.40 |  | 0.05 |  | < .001 |  | 0.39 |
| Violence _age 17_ 🡪 Violence _age 20_ |  | 0.31 |  | 0.05 |  | < .001 |  | 0.34 |
| *Cross-lagged paths* |  |  |  |  |  |  |  |  |
| Self-control _age 11_ 🡪 Tobacco use _age 13_ |  | −0.55 |  | 0.09 |  | < .001 |  | −0.22 |
| Self-control _age 11_ 🡪 Cannabis use _age 13_ |  | −0.35 |  | 0.06 |  | < .001 |  | −0.25 |
| Self-control _age 11_ 🡪 Violence _age 13_ |  | −0.37 |  | 0.06 |  | < .001 |  | −0.22 |
| Self-control _age 13_ 🡪 Tobacco use _age 15_ |  | −0.64 |  | 0.11 |  | < .001 |  | −0.17 |
| Self-control _age 13_ 🡪 Cannabis use _age 15_ |  | −0.46 |  | 0.10 |  | < .001 |  | −0.15 |
| Self-control _age 13_ 🡪 Violence _age 15_ |  | −0.20 |  | 0.06 |  | < .001 |  | −0.12 |
| Self-control _age 15_ 🡪 Tobacco use _age 17_ |  | −0.71 |  | 0.12 |  | < .001 |  | −0.17 |
| Self-control _age 15_ 🡪 Cannabis use _age 17_ |  | −0.35 |  | 0.12 |  | .003 |  | −0.09 |
| Self-control _age 15_ 🡪 Violence _age 17_ |  | −0.09 |  | 0.07 |  | .182 |  | −0.05 |
| Self-control _age 17_ 🡪 Tobacco use _age 20_ |  | −0.17 |  | 0.13 |  | .171 |  | −0.04 |
| Self-control _age 17_ 🡪 Cannabis use _age 20_ |  | −0.26 |  | 0.12 |  | .033 |  | −0.07 |
| Self-control _age 17_ 🡪 Violence _age 20_ |  | −0.10 |  | 0.06 |  | .093 |  | −0.05 |
| Tobacco use _age 13_ 🡪 Cannabis use _age 15_ |  | 0.29 |  | 0.05 |  | < .001 |  | 0.24 |
| Tobacco use _age 13_ 🡪 Self-control _age 15_ |  | −0.01 |  | 0.01 |  | .591 |  | −0.02 |
| Tobacco use _age 13_ 🡪 Violence _age 15_ |  | 0.08 |  | 0.03 |  | .007 |  | 0.12 |
| Tobacco use _age 13_ 🡪 Violence _age 17_ |  | −0.02 |  | 0.03 |  | .534 |  | −0.03 |
| Tobacco use _age 13_ 🡪 Violence _age 20_ |  | −0.03 |  | 0.03 |  | .416 |  | −0.04 |
| Tobacco use _age 15_ 🡪 Cannabis use _age 17_ |  | 0.16 |  | 0.04 |  | < .001 |  | 0.17 |
| Tobacco use _age 15_ 🡪 Self-control _age 17_ |  | −0.01 |  | 0.01 |  | .445 |  | −0.03 |
| Tobacco use _age 15_ 🡪 Violence _age 17_ |  | 0.03 |  | 0.02 |  | .135 |  | 0.07 |
| Tobacco use _age 15_ 🡪 Violence _age 20_ |  | 0.03 |  | 0.03 |  | .223 |  | 0.08 |
| Tobacco use _age 17_ 🡪 Cannabis use _age 20_ |  | 0.07 |  | 0.03 |  | .023 |  | 0.07 |
| Tobacco use _age 17_ 🡪 Self-control _age 20_ |  | −0.01 |  | 0.01 |  | .144 |  | −0.05 |
| Tobacco use _age 17_ 🡪 Violence _age 20_ |  | 0.04 |  | 0.02 |  | .025 |  | 0.09 |
| Cannabis use _age 13_ 🡪 Tobacco use _age 15_ |  | 0.01 |  | 0.07 |  | .858 |  | 0.01 |
| Cannabis use _age 13_ 🡪 Self-control _age 15_ |  | −0.01 |  | 0.02 |  | .751 |  | −0.01 |
| Cannabis use _age 13_ 🡪 Violence _age 15_ |  | −0.01 |  | 0.06 |  | .865 |  | −0.01 |
| Cannabis use _age 13_ 🡪 Violence _age 17_ |  | 0.08 |  | 0.06 |  | .220 |  | 0.07 |
| Cannabis use _age 13_ 🡪 Violence _age 20_ |  | 0.07 |  | 0.08 |  | .425 |  | 0.06 |
| Cannabis use _age 15_ 🡪 Tobacco use _age 17_ |  | −0.00 |  | 0.04 |  | .923 |  | −0.00 |
| Cannabis use _age 15_ 🡪 Self-control _age 17_ |  | −0.01 |  | 0.01 |  | .153 |  | −0.05 |
| Cannabis use _age 15_ 🡪 Violence _age 17_ |  | 0.01 |  | 0.02 |  | .679 |  | 0.02 |
| Cannabis use _age 15_ 🡪 Violence _age 20_ |  | 0.00 |  | 0.03 |  | .986 |  | 0.00 |
| Cannabis use _age 17_ 🡪 Tobacco use _age 20_ |  | 0.10 |  | 0.04 |  | .006 |  | 0.09 |
| Cannabis use _age 17_ 🡪 Self-control _age 20_ |  | 0.01 |  | 0.01 |  | .594 |  | 0.02 |
| Cannabis use _age 17_ 🡪 Violence _age 20_ |  | −0.03 |  | 0.02 |  | .217 |  | −0.06 |
| Violence _age 13_ 🡪 Tobacco use _age 15_ |  | 0.22 |  | 0.08 |  | .004 |  | 0.10 |
| Violence _age 13_ 🡪 Cannabis use _age 15_ |  | 0.10 |  | 0.07 |  | .169 |  | 0.05 |
| Violence _age 13_ 🡪 Self-control _age 15_ |  | −0.04 |  | 0.02 |  | .042 |  | −0.07 |
| Violence _age 15_ 🡪 Tobacco use _age 17_ |  | −0.08 |  | 0.06 |  | .201 |  | −0.03 |
| Violence _age 15_ 🡪 Cannabis use _age 17_ |  | −0.01 |  | 0.08 |  | .919 |  | −0.00 |
| Violence _age 15_ 🡪 Self-control _age 17_ |  | −0.01 |  | 0.02 |  | .481 |  | −0.02 |
| Violence _age 17_ 🡪 Tobacco use _age 20_ |  | 0.01 |  | 0.07 |  | .835 |  | 0.01 |
| Violence _age 17_ 🡪 Cannabis use _age 20_ |  | −0.00 |  | 0.07 |  | .981 |  | −0.00 |
| Violence _age 17_ 🡪 Self-control _age 20_ |  | −0.01 |  | 0.02 |  | .739 |  | −0.01 |

*Note.* CLPM connecting self-control, tobacco use, cannabis use, and physical violence perpetration; *n* = 1,056.

Table S2

Standardized and Unstandardized Coefficients for Cross-Sectional Covariances of Residuals (Main Model)

| *CLPM* |  | Unstandardized *r* (covariance) |  | SE |  | *p*-value |  | Standardized *r* (correlation) |
| --- | --- | --- | --- | --- | --- | --- | --- | --- |
| *Cross-sectional covariances of residuals* |  |  |  |  |  |  |  |  |
| Self-control _age 13_ with Tobacco use _age 13_ |  | −0.15 |  | 0.02 |  | < .001 |  | −0.30 |
| Self-control _age 15_ with Tobacco use _age 15_ |  | −0.11 |  | 0.02 |  | < .001 |  | −0.20 |
| Self-control _age 17_ with Tobacco use _age 17_ |  | −0.08 |  | 0.02 |  | < .001 |  | −0.17 |
| Self-control _age 20_ with Tobacco use _age 20_ |  | −0.04 |  | 0.02 |  | .013 |  | −0.08 |
| Self-control _age 13_ with Cannabis use _age 13_ |  | −0.04 |  | 0.01 |  | < .001 |  | −0.14 |
| Self-control _age 15_ with Cannabis use _age 15_ |  | −0.07 |  | 0.02 |  | < .001 |  | −0.15 |
| Self-control _age 17_ with Cannabis use _age 17_ |  | −0.07 |  | 0.02 |  | < .001 |  | −0.16 |
| Self-control _age 20_ with Cannabis use _age 20_ |  | −0.08 |  | 0.02 |  | < .001 |  | −0.19 |
| Self-control _age 13_ with Violence _age 13_ |  | −0.11 |  | 0.01 |  | < .001 |  | −0.37 |
| Self-control _age 15_ with Violence _age 15_ |  | −0.06 |  | 0.01 |  | < .001 |  | −0.25 |
| Self-control _age 17_ with Violence _age 17_ |  | −0.05 |  | 0.01 |  | < .001 |  | −0.22 |
| Self-control _age 20_ with Violence _age 20_ |  | −0.04 |  | 0.01 |  | < .001 |  | −0.20 |
| Cannabis use _age 13_ with Tobacco use _age 13_ |  | 0.37 |  | 0.06 |  | < .001 |  | 0.51 |
| Cannabis use _age 15_ with Tobacco use _age 15_ |  | 0.90 |  | 0.07 |  | < .001 |  | 0.50 |
| Cannabis use _age 17_ with Tobacco use _age 17_ |  | 0.63 |  | 0.07 |  | < .001 |  | 0.34 |
| Cannabis use _age 20_ with Tobacco use _age 20_ |  | 0.53 |  | 0.07 |  | < .001 |  | 0.29 |
| Cannabis use _age 13_ with Violence _age 13_ |  | 0.12 |  | 0.03 |  | < .001 |  | 0.25 |
| Cannabis use _age 15_ with Violence _age 15_ |  | 0.08 |  | 0.03 |  | .009 |  | 0.10 |
| Cannabis use _age 17_ with Violence _age 17_ |  | 0.06 |  | 0.04 |  | .114 |  | 0.07 |
| Cannabis use _age 20_ with Violence _age 20_ |  | 0.05 |  | 0.04 |  | .226 |  | 0.06 |
| Violence _age 13_ with Tobacco use _age 13_ |  | 0.28 |  | 0.04 |  | < .001 |  | 0.33 |
| Violence _age 15_ with Tobacco use _age 15_ |  | 0.19 |  | 0.04 |  | < .001 |  | 0.19 |
| Violence _age 17_ with Tobacco use _age 17_ |  | 0.13 |  | 0.03 |  | < .001 |  | 0.14 |
| Violence _age 20_ with Tobacco use _age 20_ |  | 0.07 |  | 0.03 |  | .039 |  | 0.07 |

*Note.* CLPM connecting self-control, tobacco use, cannabis use, and physical violence perpetration; *n* = 1,056.

Table S3

Standardized and Unstandardized Coefficients for Control Variables (Main Model)

| CLPM |  | Unstandardized beta |  | SE |  | *p*-value |  | Standardized beta (β) |
| --- | --- | --- | --- | --- | --- | --- | --- | --- |
| *Control variables* |  |  |  |  |  |  |  |  |
| Sex 🡪 Self-control _age 13_ |  | −0.06 |  | 0.03 |  | .029 |  | −0.06 |
| Sex 🡪 Self-control _age 17_ |  | −0.06 |  | 0.02 |  | .011 |  | −0.07 |
| Sex 🡪 Self-control _age 20_ |  | −0.06 |  | 0.03 |  | .013 |  | −0.08 |
| Sex 🡪 Tobacco use _age 13_ |  | 0.17 |  | 0.07 |  | .020 |  | 0.07 |
| Sex 🡪 Tobacco use _age 15_ |  | −0.18 |  | 0.09 |  | .048 |  | −0.05 |
| Sex 🡪 Cannabis use _age 13_ |  | 0.22 |  | 0.04 |  | < .001 |  | 0.17 |
| Sex 🡪 Cannabis use _age 15_ |  | 0.18 |  | 0.08 |  | .025 |  | 0.06 |
| Sex 🡪 Cannabis use _age 17_ |  | 0.36 |  | 0.09 |  | < .001 |  | 0.11 |
| Sex 🡪 Cannabis use _age 20_ |  | 0.33 |  | 0.09 |  | < .001 |  | 0.10 |
| Sex 🡪 Violence _age 13_ |  | 0.53 |  | 0.05 |  | < .001 |  | 0.33 |
| Sex 🡪 Violence _age 15_ |  | 0.28 |  | 0.04 |  | < .001 |  | 0.17 |
| Sex 🡪 Violence _age 17_ |  | 0.36 |  | 0.05 |  | < .001 |  | 0.22 |
| Sex 🡪 Violence _age 20_ |  | 0.18 |  | 0.05 |  | .001 |  | 0.12 |
| Migration background 🡪 Cannabis use _age 15_ |  | −0.22 |  | 0.08 |  | .005 |  | −0.08 |
| Migration background 🡪 Cannabis use _age 17_ |  | −0.33 |  | 0.09 |  | < .001 |  | −0.10 |
| Migration background 🡪 Cannabis use _age 20_ |  | −0.24 |  | 0.09 |  | .007 |  | −0.07 |
| Migration background 🡪 Violence _age 13_ |  | 0.10 |  | 0.05 |  | .030 |  | 0.06 |
| Migration background 🡪 Violence _age 17_ |  | 0.09 |  | 0.05 |  | .052 |  | 0.05 |
| SES 🡪 Self-control _age 15_ |  | 0.00 |  | 0.00 |  | .024 |  | 0.06 |
| SES 🡪 Self-control _age 17_ |  | 0.00 |  | 0.00 |  | .009 |  | 0.06 |
| SES 🡪 Tobacco use _age 20_ |  | −0.01 |  | 0.00 |  | < .001 |  | −0.09 |
| SES 🡪 Cannabis use _age 15_ |  | 0.01 |  | 0.00 |  | < .001 |  | 0.13 |
| SES 🡪 Cannabis use _age 17_ |  | 0.01 |  | 0.00 |  | < .001 |  | 0.10 |
| SES 🡪 Violence _age 13_ |  | −0.00 |  | 0.00 |  | < .001 |  | −0.11 |
| SES 🡪 Violence _age 15_ |  | −0.00 |  | 0.00 |  | .002 |  | −0.08 |
| SES 🡪 Violence _age 17_ |  | −0.00 |  | 0.00 |  | .018 |  | −0.07 |
| SES 🡪 Violence _age 20_ |  | −0.00 |  | 0.00 |  | .039 |  | −0.06 |

*Note.* CLPM connecting self-control, tobacco use, cannabis use, and physical violence perpetration; *n* = 1,056.

## Sensitivity Analysis

Table S4

Correlations, Means, and Standard Deviations of Study Variables (Sensitivity Analysis)

| Variables | 1 | 2 | 3 | 4 | 5 | 6 | 7 | 8 | 9 | 10 | 11 | 12 | 13 | 14 | 15 | 16 | 17 | n | *M (SD)* |
| --- | --- | --- | --- | --- | --- | --- | --- | --- | --- | --- | --- | --- | --- | --- | --- | --- | --- | --- | --- |
| 1. Self-control _age11_ | 1 |  |  |  |  |  |  |  |  |  |  |  |  |  |  |  |  | 1056 | 3.05(.47) |
| 2. Self-control _age13_ | .44^***^ | 1 |  |  |  |  |  |  |  |  |  |  |  |  |  |  |  | 979 | 2.80(.47) |
| 3. Self-control _age15_ | .33^***^ | .52^***^ | 1 |  |  |  |  |  |  |  |  |  |  |  |  |  |  | 1035 | 2.73(.44) |
| 4. Self-control _age17_ | .32^***^ | .43^***^ | .62^***^ | 1 |  |  |  |  |  |  |  |  |  |  |  |  |  | 947 | 2.79(.42) |
| 5. Self-control _age20_ | .23^***^ | .33^***^ | .46^***^ | .60^***^ | 1 |  |  |  |  |  |  | . |  |  |  |  |  | 879 | 2.94(.42) |
| 6. Alcohol use _age 13_ | −.24^***^ | −.28^***^ | −.16^***^ | −.14^***^ | −.09^**^ | 1 |  |  |  |  |  |  |  |  |  |  |  | 974 | 1.61(.99) |
| 7. Alcohol use _age 15_ | −.25^***^ | −.31^***^ | −.26^***^ | −.23^***^ | −.12^***^ | .42^***^ | 1 |  |  |  |  |  |  |  |  |  |  | 1030 | 2.58(1.35) |
| 8. Alcohol use _age 17_ | −.23^***^ | −.19^***^ | −.18^***^ | −.25^***^ | −.11^**^ | .28^***^ | .55^***^ | 1 |  |  |  |  |  |  |  |  |  | 948 | 3.49(1.38) |
| 9. Alcohol use _age 20_ | −.12^***^ | −.13^***^ | −.08^*^ | −.14^***^ | −.10^**^ | .16^***^ | .37^***^ | .61^***^ | 1 |  |  |  |  |  |  |  |  | 878 | 3.95(1.29) |
| 10. Cannabis use _age13_ | −.27^***^ | −.25^***^ | −.17^***^ | −.14^***^ | −.15^***^ | .46^***^ | .22^***^ | .13^***^ | .05 | 1 |  |  |  |  |  |  |  | 968 | 1.18(.67) |
| 11. Cannabis use _age15_ | −.22^***^ | −.32^***^ | −.28^***^ | −.23^***^ | −.17^***^ | .39^***^ | .55^***^ | .34^***^ | .22^***^ | .38^***^ | 1 |  |  |  |  |  |  | 1027 | 1.93(1.46) |
| 12. Cannabis use _age17_ | −.26^***^ | −.29^***^ | −.24^***^ | −.29^***^ | −.18^***^ | .29^***^ | .45^***^ | .50^***^ | .34^***^ | .29^***^ | .57^***^ | 1 |  |  |  |  |  | 940 | 2.54(1.67) |
| 13. Cannabis use _age20_ | −.20^***^ | −.25^***^ | −.20^***^ | −.24^***^ | −.28^***^ | .23^***^ | .33^***^ | .38^***^ | .36^***^ | .21^***^ | .40^***^ | .61^***^ | 1 |  |  |  |  | 878 | 2.56(1.68) |
| 14. Violence _age13_ | −.26^***^ | −.44^***^ | −.30^***^ | −.26^***^ | −.24^***^ | .33^***^ | .18^***^ | .10^**^ | .08^*^ | .33^***^ | .26^***^ | .23^***^ | .22^***^ | 1 |  |  |  | 982 | −.01(.81) |
| 15. Violence _age15_ | −.19^***^ | −.34^***^ | −.38^***^ | −.29^***^ | −.25^***^ | .20^***^ | .21^***^ | .06^*^ | .00 | .24^***^ | .26^***^ | .20^***^ | .17^***^ | .50^***^ | 1 |  |  | 1037 | −.03(.79) |
| 16. Violence _age17_ | −.19^***^ | −.21^***^ | −.23^***^ | −.33^***^ | −.23^***^ | .17^***^ | .11^***^ | .09^**^ | .01 | .21^***^ | .20^***^ | .19^***^ | .14^***^ | .34^***^ | .51^***^ | 1 |  | 954 | −.04(.81) |
| 17. Violence _age20_ | −.11^**^ | −.09^**^ | −.17^***^ | −.21^***^ | −.31^***^ | .10^**^ | .08^*^ | −.02 | .01 | .18^***^ | .14^***^ | .08^*^ | .13^***^ | .21^***^ | .36^***^ | .41^***^ | 1 | 880 | −.02(.74) |
| *Controls* |  |  |  |  |  |  |  |  |  |  |  |  |  |  |  |  |  |  |  |
| Sex (1) | −.17^***^ | −.14^***^ | −.08^**^ | −.11^***^ | −.14^***^ | .16^***^ | .06 | .09^**^ | .10^**^ | .21^***^ | .18^***^ | .17^***^ | .19^***^ | .36^***^ | .32^***^ | .35^***^ | .23^***^ | 1056 | 51% |
| Migration background (1) | .11^***^ | .05 | −.02 | −.03 | −.05 | −.07^*^ | −.18^***^ | −.30^***^ | −.26^***^ | −.03 | −.15^***^ | −.19^***^ | −.17^***^ | .08^*^ | .07^*^ | .10^**^ | .11^**^ | 1056 | 45% |
| Family SES | −.08^**^ | −.02 | .06 | .10^**^ | .11^**^ | .03 | .22^***^ | .32^***^ | .31^***^ | −.01 | .15^***^ | .19^***^ | .15^***^ | −.13^***^ | −.12^***^ | −.14^***^ | −.14^***^ | 1056 | 47.81(19.7) |

*Note. N* = 1,056; *M* = mean, *SD* = standard deviation; **p* < .05, ***p* < .01, ****p* < .001. Missing = pairwise. Dummy coding: Sex assigned at birth  (1) = male; parental migration background (1) = both parents born abroad.


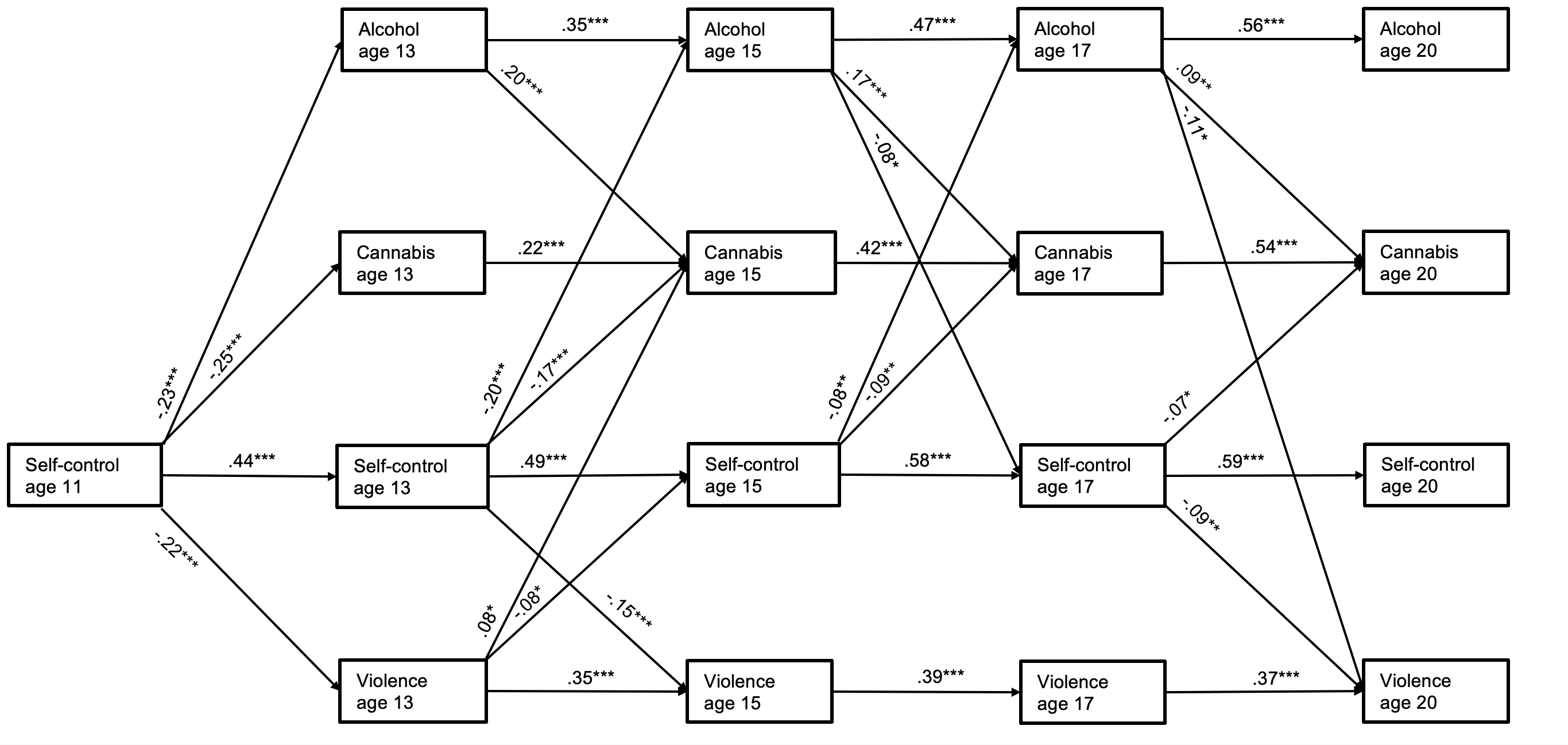


**Fig. S2** Fit estimates of CLPM (sensitivity analysis): $\chi$^2^ (73) = 168.607, *p* <.001; CFI = .984; TLI = .960; RMSEA = .035; SRMR = .029. Cross-lagged panel model connecting self-control, alcohol use, cannabis use, and physical violence perpetration; *n* = 1,056, **p* < .05, ***p* < .01, ****p* < .001. Significant standardized path coefficients. For the sake of simplicity, cross-sectional covariances of residuals are omitted. The model is adjusted for sex assigned at birth, parental migration background, and family SES

Table S5

Autoregressive and Cross-Lagged Paths Among Self-control, Alcohol Use, Cannabis Use, and Physical Violence Perpetration (Sensitivity Analysis)

| *Outcome variable at age 13* | | | | | | | |  | *Outcome variable at age 15* | | | | | | | |
| --- | --- | --- | --- | --- | --- | --- | --- | --- | --- | --- | --- | --- | --- | --- | --- | --- |
|  | *Cannabis use* |  | *Alcohol use* |  | *Self-control* |  | *Violence* |  |  | *Cannabis use* |  | *Alcohol use* |  | *Self-control* |  | *Violence* |
|  | *β* (SE) |  | *β* (SE) |  | *β* (SE) |  | *β* (SE) |  |  | *β* (SE) |  | *β* (SE) |  | *β* (SE) |  | *β* (SE) |
| *Predictors* |  |  |  |  |  |  |  |  |  |  |  |  |  |  |  |  |
|  |  |  |  |  |  |  |  |  |  |  |  |  |  |  |  |  |
|  |  |  |  |  |  |  |  |  |  |  |  |  |  |  |  |  |
|  |  |  |  |  |  |  |  |  | Cannabis use _age13_ | .22(.09)^***^ |  | .01(.07) |  | −.03(.02) |  | .05(.06) |
| Self-control _age11_ | −.25(.06)^***^ |  | −.23(.07)^***^ |  | .44(.03)^***^ |  | −.22(.06)^***^ |  | Alcohol use _age 13_ | .20(.06)^***^ |  | .35(.04)^***^ |  | .02(.01) |  | −.01(.03) |
|  |  |  |  |  |  |  |  |  | Self-control _age13_ | −.17(.10)^***^ |  | −.20(.09)^***^ |  | .49(.03)^***^ |  | −.15(.06)^***^ |
|  |  |  |  |  |  |  |  |  | Violence _age13_ | .08(.07)^*^ |  | −.00(.06) |  | −.08(.02)^*^ |  | .35(.05)^***^ |
| *Controls* |  |  |  |  |  |  |  |  | *Controls* |  |  |  |  |  |  |  |
| Sex (1) | .17(.04)^***^ |  | .11(.06)^***^ |  | −.06(.03)^*^ |  | .33(.05)^***^ |  | Sex (1) |  |  |  |  |  |  | .17(.04)^***^ |
| Migration background (1) |  |  |  |  |  |  | .07(.05)^*^ |  | Migration background (1) | −.07(.09)^*^ |  | −.07(.08)^*^ |  |  |  |  |
| Family SES |  |  |  |  |  |  | −.11(.00)^***^ |  | Family SES | .12(.00)^***^ |  | .17(.00)^***^ |  | .06(.00)^*^ |  | −.08(.00)^**^ |
| *Outcome variable at age 17* | | | | | | | |  | *Outcome variable at age 20* | | | | | | | |
|  | *Cannabis use* |  | *Alcohol use* |  | *Self-control* |  | *Violence* |  |  | *Cannabis use* |  | *Alcohol use* |  | *Self-control* |  | *Violence* |
|  | *β* (SE) |  | *β* (SE) |  | *β* (SE) |  | *β* (SE) |  |  | *β* (SE) |  | *β* (SE) |  | *β* (SE) |  | *β* (SE) |
| Cannabis use _age13_ |  |  |  |  |  |  | .05(.06) |  | Cannabis use _age13_ |  |  |  |  |  |  | .06(.08) |
| Cannabis use _age15_ | .42(.04)^***^ |  | .02(.03) |  | −.03(.01) |  | .04(.02) |  | Cannabis use _age15_ |  |  |  |  |  |  | .02(.02) |
|  |  |  |  |  |  |  |  |  | Cannabis use _age17_ | .54(.04)^***^ |  | .04(.02) |  | −.02(.01) |  | −.00(.02) |
| Alcohol use _age 13_ |  |  |  |  |  |  | .02(.04) |  | Alcohol use _age 13_ |  |  |  |  |  |  | −.02(.03) |
| Alcohol use _age 15_ | .17(.04)^***^ |  | .47(.03)^***^ |  | −.08(.01)^*^ |  | −.00(.02) |  | Alcohol use _age 15_ |  |  |  |  |  |  | .07(.03) |
|  |  |  |  |  |  |  |  |  | Alcohol use _age17_ | .09(.04)^**^ |  | .56(.04)^***^ |  | .05(.01) |  | −.11(.02)^*^ |
| Self-control _age15_ | −.09(.12)^**^ |  | −.08(.10)^**^ |  | .58(.03)^***^ |  | −.05(.07) |  | Self-control _age17_ | −.07(.12)^*^ |  | −.01(.10) |  | .59(.03)^***^ |  | −.09(.06)^**^ |
| Violence _age15_ | .02(.08) |  | −.06(.06) |  | −.02(.02) |  | .39(.05)^***^ |  | Violence _age17_ | .01(.07) |  | −.05(.06) |  | −.02(.02) |  | .37(.05)^***^ |
| *Controls* |  |  |  |  |  |  |  |  | *Controls* |  |  |  |  |  |  |  |
| Sex (1) | .09(.09)^**^ |  | .06(.08)^*^ |  | −.06(.02)^*^ |  | .21(.05)^***^ |  | Sex (1) | .09(.09)^**^ |  | .07(.07)^*^ |  | −.07(.03)^*^ |  | .09(.05)^**^ |
| Migration background (1) | −.09(.10)^**^ |  | −.15(.08)^***^ |  |  |  | .06(.05)^*^ |  | Migration background (1) |  |  |  |  |  |  |  |
| Family SES | .06(.00) |  | .14(.00)^***^ |  | .08(.00)^**^ |  | −.08(.00)^*^ |  | Family SES |  |  | .11(.00)^***^ |  |  |  |  |

*Note.* CLPM connecting self-control, alcohol use, cannabis use, and physical violence perpetration; *n* = 1,056; **p* < .05, ***p* < .01, ****p* < .001. Control variables *p* < .1 included in model. Dummy coding: sex assigned at birth (1) = male; parental migration background (1) = both parents born abroad.

Table S6

Standardized and Unstandardized Coefficients for Cross-Sectional Covariances of Residuals (Sensitivity Analysis)

| *CLPM* |  | Unstandardized  *r* (covariance) |  | SE |  | *p*-value |  | Standardized  *r* (correlation) |
| --- | --- | --- | --- | --- | --- | --- | --- | --- |
| *Cross-sectional covariances of residuals* |  |  |  |  |  |  |  |  |
| Self-control _age 13_ with Violence _age 13_ |  | −0.11 |  | 0.01 |  | < .001 |  | −0.37 |
| Self-control _age 15_ with Violence _age 15_ |  | −0.06 |  | 0.01 |  | < .001 |  | −0.25 |
| Self-control _age 17_ with Violence _age 17_ |  | −0.05 |  | 0.01 |  | < .001 |  | −0.22 |
| Self-control _age 20_ with Violence _age 20_ |  | −0.05 |  | 0.01 |  | < .001 |  | −0.21 |
| Self-control _age 13_ with Cannabis use _age 13_ |  | −0.04 |  | 0.01 |  | < .001 |  | −0.14 |
| Self-control _age 15_ with Cannabis use _age 15_ |  | −0.07 |  | 0.02 |  | < .001 |  | −0.15 |
| Self-control _age 17_ with Cannabis use _age 17_ |  | −0.07 |  | 0.02 |  | < .001 |  | −0.16 |
| Self-control _age 20_ with Cannabis use _age 20_ |  | −0.09 |  | 0.02 |  | < .001 |  | −0.20 |
| Self-control _age 13_ with Alcohol use _age 13_ |  | −0.08 |  | 0.01 |  | < .001 |  | −0.20 |
| Self-control _age 15_ with Alcohol use _age 15_ |  | −0.07 |  | 0.02 |  | < .001 |  | −0.16 |
| Self-control _age 17_ with Alcohol use _age 17_ |  | −0.07 |  | 0.01 |  | < .001 |  | −0.19 |
| Self-control _age 20_ with Alcohol use _age 20_ |  | −0.02 |  | 0.01 |  | .090 |  | −0.06 |
| Cannabis use _age 13_ with Alcohol use _age 13_ |  | 0.25 |  | 0.04 |  | < .001 |  | 0.41 |
| Cannabis use _age 15_ with Alcohol use _age 15_ |  | 0.61 |  | 0.06 |  | < .001 |  | 0.42 |
| Cannabis use _age 17_ with Alcohol use _age 17_ |  | 0.46 |  | 0.05 |  | < .001 |  | 0.32 |
| Cannabis use _age 20_ with Alcohol use _age 20_ |  | 0.23 |  | 0.05 |  | < .001 |  | 0.17 |
| Cannabis use _age 13_ with Violence _age 13_ |  | 0.11 |  | 0.03 |  | < .001 |  | 0.25 |
| Cannabis use _age 15_ with Violence _age 15_ |  | 0.10 |  | 0.03 |  | .001 |  | 0.12 |
| Cannabis use _age 17_ with Violence _age 17_ |  | 0.07 |  | 0.04 |  | .089 |  | 0.07 |
| Cannabis use _age 20_ with Violence _age 20_ |  | 0.05 |  | 0.04 |  | .204 |  | 0.06 |
| Violence _age 13_ with Alcohol use _age 13_ |  | 0.19 |  | 0.03 |  | < .001 |  | 0.27 |
| Violence _age 15_ with Alcohol use _age 15_ |  | 0.11 |  | 0.03 |  | < .001 |  | 0.15 |
| Violence _age 17_ with Alcohol use _age 17_ |  | 0.06 |  | 0.03 |  | .027 |  | 0.08 |
| Violence _age 20_ with Alcohol use _age 20_ |  | 0.03 |  | 0.04 |  | .510 |  | 0.04 |

*Note.* CLPM connecting self-control, alcohol use, cannabis use, and physical violence perpetration; *n* = 1,056.

Table S7

Unstandardized Estimates of Indirect Effects, Standard Errors, and 95% Bias-Corrected Bootstrap Confidence Intervals (Sensitivity Analysis)

|  | |  |  | *Confidence intervals* | |
| --- | --- | --- | --- | --- | --- |
|  | Estimate | SE | | Lower | Upper |
| A_age 13_🡪 SC_age 15_ 🡪 V_age 17_ 🡪 V_age 20_ | −.000 | .001 | | −.002 | .000 |
| A_age 13_🡪 SC_age 15_ 🡪 V_age 17_ | −.001 | .002 | | −.006 | .002 |
| A_age 13_🡪 A_age 15_ 🡪 SC_age 17_ 🡪 V_age 20_ | .002 | .001 | | .000 | .005 |
| A_age 15_ 🡪 SC_age 17_ 🡪 V_age 20_ | **.004** | **.002** | | **.001** | **.010** |
| A_age 13_🡪 SC_age 15_ 🡪 SC_age 17_ 🡪 V_age 20_ | −.001 | .001 | | −.004 | .002 |
| C_age 13_🡪 SC_age 15_ 🡪 V_age 17_ 🡪 V_age 20_ | .001 | .001 | | −.001 | .004 |
| C_age 13_🡪 SC_age 15_ 🡪 V_age 17_ | .002 | .003 | | −.002 | .011 |
| C_age 13_🡪 C_age 15_ 🡪 SC_age 17_ 🡪 V_age 20_ | .001 | .001 | | .000 | .003 |
| C_age 15_ 🡪 SC_age 17_ 🡪 V_age 20_ | .001 | .002 | | −.001 | .006 |
| C_age 13_🡪 SC_age 15_ 🡪 SC_age 17_ 🡪 V_age 20_ | .002 | .002 | | −.002 | .007 |

*Note.* A = alcohol use, C = cannabis use, SC = self-control, V = physical violence perpetration. Bold typeface displays regression estimates where the confidence interval does not include 0 (i.e., significance); *n* = 1,056. Direct associations included cross-lagged paths from alcohol and cannabis use at age 13 to physical violence perpetration at age 17 and age 20 as well as from alcohol and cannabis use at age 15 to physical violence perpetration at age 20.

# References

1. Grasmick HG, Tittle CR, Bursik Jr RJ, Arneklev BJ (1993) Testing the core empirical implications of Gottfredson and Hirschi’s general theory of crime. J Res Crime Delinq 30:5–29. <https://doi.org/10.1177/0022427893030001002>

2. Longshore D, Rand ST, Stein JA (1996) Self-control in a criminal sample: An examination of construct validity. Criminology 34:209–228. <https://doi.org/10.1111/j.1745-9125.1996.tb01203.x>

3. Tremblay RE, Loeber R, Gagnon C, Charlebois P, Larivée S, LeBlanc M (1991) Disruptive boys with stable and unstable high fighting behavior patterns during junior elementary school. J Abnorm Child Psychol 19:285–300. <https://doi.org/10.1007/BF00911232>

4. Olweus D (1993) Victimization by peers: Antecedents and long‐term outcomes. In: Rubin KH, Asendorpf JB (eds) Social withdrawal, inhibition, and shyness in childhood. Psychology Press, New York, pp 315–341. <https://doi.org/10.4324/9781315799544>
